# Minimally Invasive Treatment for Nonunion in an Unusual Pattern of Neuropathic Calcaneal Fracture: A Case Report

**DOI:** 10.7759/cureus.21718

**Published:** 2022-01-29

**Authors:** Rakesh Goyal, Ajay Gupta, Nishant Bhatia, Akash Goel, Ayush Gupta

**Affiliations:** 1 Department of Orthopaedics, Sports Injury Centre, Vardhman Mahavir Medical College and Safdarjung Hospital, New Delhi, IND; 2 Department of Orthopaedic Surgery, Maulana Azad Medical College and Associated Lok Nayak Hospital, New Delhi, IND; 3 Department of Orthopaedics, Himalayan Institute of Medical Science, Dehradun, IND

**Keywords:** nonunion, neuropathic, minimally invasive, keyhole, calcaneum

## Abstract

Neuropathic arthropathy is the painless destruction of weight-bearing bones and joints that is caused by a neurosensory deficit. Minimally displaced neuropathic fractures may be managed non-operatively. However, larger displacements often require surgical fixation. Nonunion is a rare entity in calcaneal fractures, and its occurrence in a neuropathic setting is an even rarer scenario. An unusual clinical scenario means there is a paucity of literature to guide the optimum treatment. Here we describe a patient with neuropathic arthropathy due to meningomyelocele in childhood, presenting with neuropathic nonunion of the calcaneum, managed with a minimally invasive surgical approach using an arthroscopic burr and fixation with percutaneous screws. Despite the high risk of complications in the operative treatment of neuropathic fractures, good functional results and successful limb salvage may be achieved with minimally invasive techniques.

## Introduction

Neuropathic arthropathy is the painless destruction of weight-bearing bones and joints caused by a neurosensory deficit [1]. The neurosensory symptoms among these patients are usually caused by pathology in the spinal cord, like its injury, tabes dorsalis, meningomyelocele, or in the peripheral nerves, as in cases of diabetes mellitus, peripheral nerve injuries, or tumors [2]. The most common causes of neuropathic arthropathy described in the literature are diabetes mellitus, tabes, syringomyelia, and leprosy [3].

Neuropathic arthropathy of the ankle and foot is commonly associated with spontaneous calcaneal fractures [3]. There is usually no history of trauma, or it could be associated with some trivial trauma [3]. Due to the absence of protective sensations, these fractures are often diagnosed late, when a deformity has developed and the patient notices the abnormal foot alignment. Treatment in such a case may require surgical management to obtain a shoeable foot, but surgery in Charcot’s arthropathy carries a high risk of complications like wound dehiscence, infection, loss of implant purchase, etc [4]. We describe a patient with neuropathic arthropathy due to meningomyelocele presenting with an unusual pattern of spontaneous calcaneal fracture that failed to heal with conservative management and was subsequently treated as a nonunion. We utilized a minimally invasive technique to minimize the risk of wound complications in this neuropathic scenario, and we were successful in obtaining fracture union along with a good outcome. 

## Case presentation

A 22-year-old male patient presented to us with swelling in his right heel. He was a known case of lumbosacral meningomyelocele with a low-lying tethered cord and had a partial sensory loss in the L4-L5 dermatomal distribution.

There was broadening of the heel with non-tender bony hard swelling all around the calcaneum. Radiographs showed an extra-articular undisplaced calcaneal fracture which was treated with a non-weight-bearing below-knee plaster cast for three months, followed by an ankle-foot orthosis (AFO).

**Figure 1:**
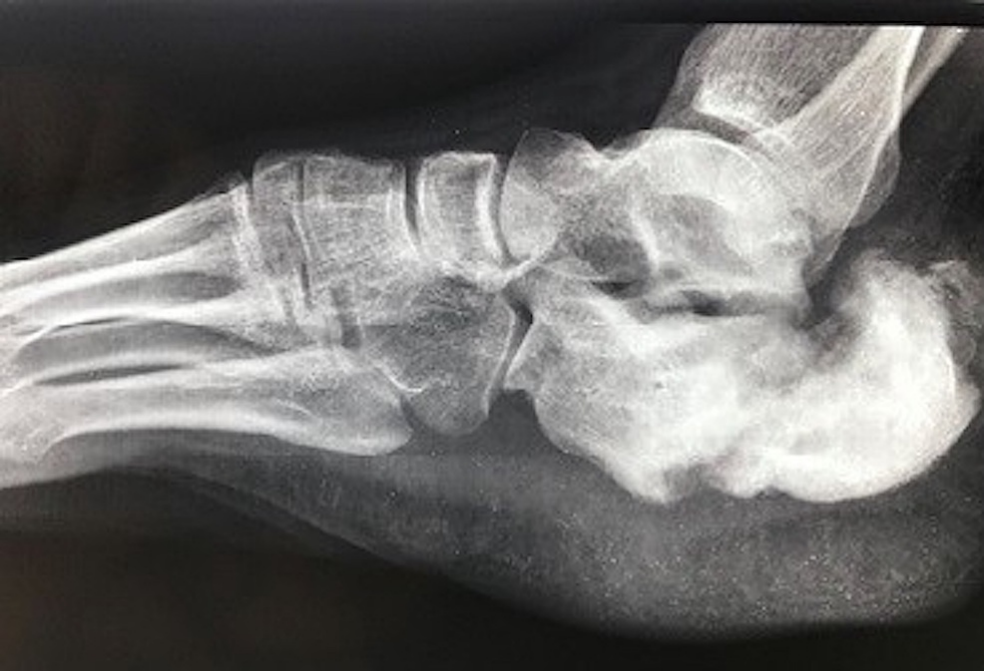
Radiograph of the right foot showing a displaced fracture of the calcaneum with irregular and sclerosed fracture margins.

**Figure 2:**
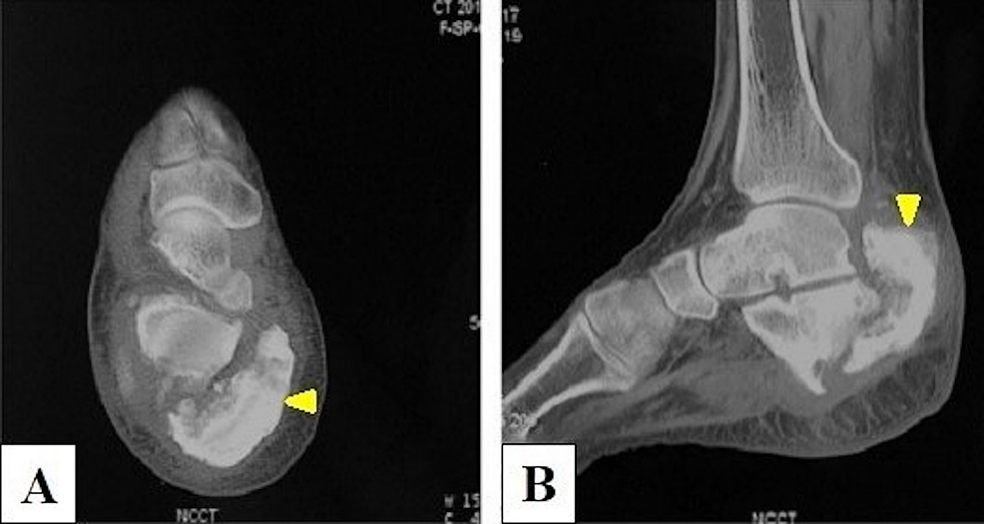
Computed tomography of the heel region. (A) Axial view showing the displaced posteromedial fracture fragment bearing the whole of the calcaneal tuberosity (yellow arrowhead). (B) Lateral view showing posterosuperior displacement of tuberosity fragment (yellow arrowhead).

Follow-up radiographs (Figure 1) and computed tomography (CT) of the right calcaneum (Figure 2) at six months demonstrated nonunion and severely displaced fragments with irregular and sclerosed fracture margins. Axial view CT showed a big posteromedial fracture fragment bearing whole of calcaneal tuberosity (Figure 2A) and attachment of tendoachilles having gross overriding with posterosuperior displacement on lateral view (Figure 2B).

**Figure 3:**
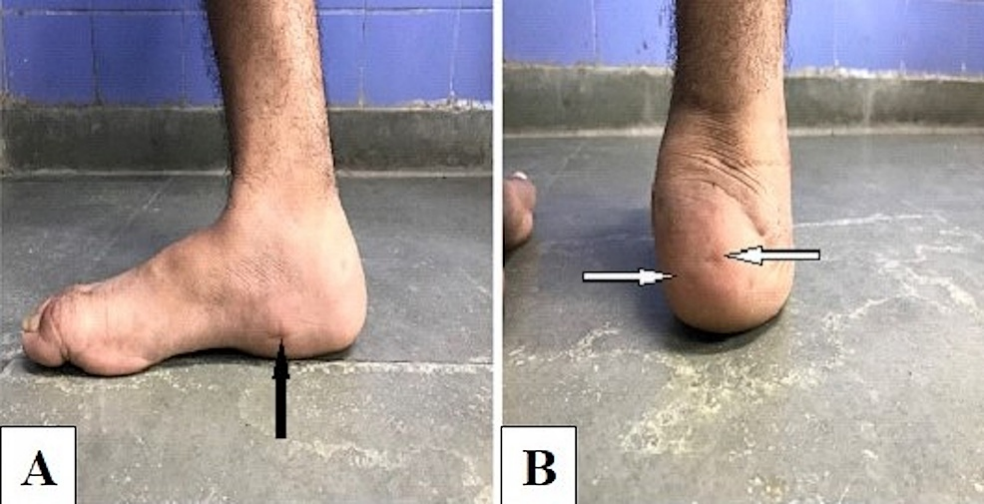
Clinical images. (A) Incision site for arthroscopic burr and shaver (black arrow). (B) Sites for percutaneous screw insertion (white arrows).

**Figure 4:**
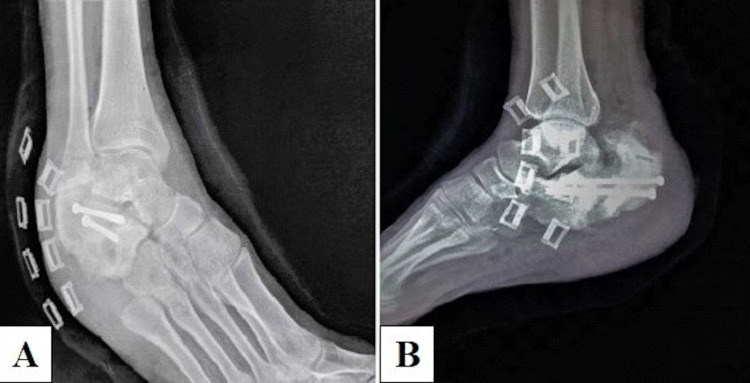
Immediate postoperative radiographs showing acceptable reduction and adequate compression. (A) Oblique view. (B) Lateral view.

The patient was operated in spinal anesthesia under tourniquet control. Nonunion site was approached through a stab incision made over the medial side of the heel region (Figure 3A). An arthroscopic burr and shaver were inserted into the nonunion site, confirmed with an image intensifier, and fracture margins were carefully freshened to avoid damage to soft tissues. Thereafter, percutaneous K-wires were inserted into the fracture fragments and utilized as joysticks to reduce the fracture. Two 4 mm cannulated cancellous screws were inserted percutaneously (Figure 3B) under fluoroscopic guidance and a compression dressing was given. X-rays showed acceptable reduction and adequate compression (Figure 4).

**Figure 5:**
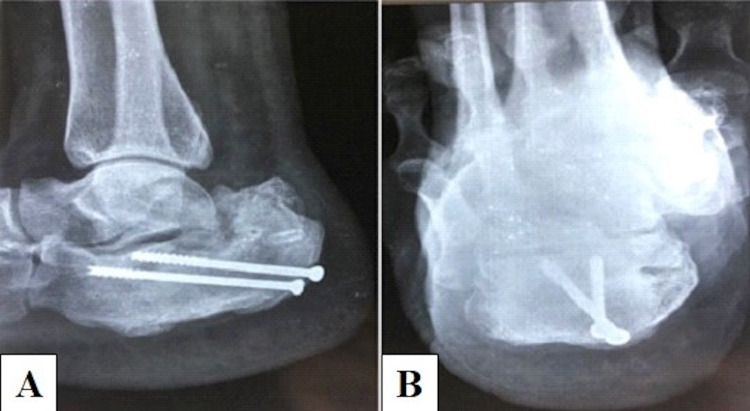
Follow-up radiographs (weight-bearing) showing union at the fracture site. (A) Lateral view. (B) Axial view.

The postoperative period was uneventful. Ankle range of motion was started immediately post-surgery. Partial weight-bearing was allowed at three months post-surgery and full weight-bearing at five months after clinical and radiological union of the fracture (Figure 5). At the last follow-up of 20 months, the patient was able to walk with a near normal gait with no deformity. The range of motion at the ankle joint was normal. He was comfortably able to do his routine outdoor activities on shoeable feet.

## Discussion

Both physiological and mechanical factors contribute to the Charcot calcaneus. There is autonomic neuropathy, which causes increased blood flow and hence bone resorption in the affected area [2]. Due to the associated sensory neuropathy and loss of protective sensation, stress injuries develop as a result of violent steppage. Symptoms related to bone stress injuries may be absent in such patients. Typically, there is an absence of load-related pain, instead there is load-related swelling, which was the scene in the present case. With continued overuse or acute trauma, stress injuries may progress to complete fractures [4].

Three main patterns of neuropathic calcaneal fractures have been described in previous reports: posterior avulsion, joint depression, and anterior process fracture [3]. A posterior avulsion fracture of the calcaneal tuberosity may cause proximal migration of the tuberosity fragment and result in ulceration of the overlying skin. The depression fracture is analogous to the traumatic joint depression variant described by Essex-Lopresti [5]. The talus settles into the calcaneus, leading to both coronal and sagittal plane deformities, which can worsen over time, leading to hindfoot ulceration. An anterior process fracture can result either from compression by an abduction force or avulsion by an adduction force. Avulsion fractures cause minimal deformity, but compression fractures can cause significant talonavicular joint subluxation, leading to potential rocker bottom formation and skin ulceration [3,6].

The fracture pattern in the present case (Figures 1, 2) is unusual in that it looks like a tuberosity avulsion fracture, but the fracture line is seen to extend obliquely in the transverse plane from posterosuperior to anteriorly and distally, all the way to the inferior surface of the calcaneum (unlike the avulsion pattern in which the fracture line is usually horizontal and the avulsed fragment displaces superiorly, hinging anteriorly just behind the posterior facet or sometimes at the posterior facet if the fracture is intra-articular). It appears as if the whole calcaneal tuberosity fractured and sheared away from the remaining bone posterosuperiorly, probably due to an obliquely upward directed force (in the direction parallel to the fracture line) striking the posterior part of the heel from below. To the best of our knowledge, no literature describes this type of extra-articular fracture. A significant gap at the fracture site and sclerosed margins indicated that the fracture had progressed to nonunion (Figure 2).

Treatment goals for neuropathic fractures include maintenance of plantigrade foot alignment, prevention of ulceration, and achieving a braceable foot. Nonoperative treatment is recommended in most cases, which consists of initial control of the acute Charcot process by limb elevation, non-weightbearing, compression bandaging, and total contact casting. These are continued until the acute (Eichenholtz stage I) inflammation like phase has resolved [7]. Long-term orthotic use or bracing is then begun with an ankle-foot orthosis (AFO) or Charcot restraint orthotic walker (CROW) device [3,8]. Recent reports recommend surgical treatment when nonoperative treatment fails to maintain a stable braceable foot or prevent recurrent ulceration. However, there is a high risk of complications associated with surgical treatment, including infection, wound dehiscence, nonunion, malunion, neurovascular injury, and amputation. Also, in neuropathic fractures, fixation without arthrodesis is ineffective due to loss of implant purchase [3,7,8].

Minimally displaced tuberosity avulsion fractures are treated with cast immobilization in equinus. Surgical fixation with cannulated screws has been recommended for larger displacements [3]. Surgical treatment was deemed necessary in this case to prevent complete hindfoot collapse, proximal migration of tuberosity fragment, and to reduce heel width so as to achieve a shoeable foot.

Neuropathic nonunion is a rare entity in calcaneal fractures, which means there is a paucity of literature to guide the optimum treatment. Suitable options include open reduction and internal fixation with screw/plate fixation with or without bone grafting, corrective osteotomy with bone grafting, and subtalar arthrodesis [9]. However, arthrodesis is the only logical option. The major challenge in managing this case was that freshening of margins and bone grafting by an open approach posed a great risk of complications. A minimally invasive approach would have a lower risk of wound complications, but there would still be a significant chance of failure of treatment as freshening may not be adequate. After discussing with the patient, it was decided to proceed with a minimally invasive approach for the same. The procedure was performed through a keyhole incision on the superior aspect of the posteromedial portion of the heel. An arthroscopic shaver was utilized to clean the fracture site of any soft tissue debris, and then the bony margins were freshened using an arthroscopic burr. Such use of arthroscopic shaver and burr is prevalent in arthroscopic ankle and subtalar arthrodesis but not in managing calcaneal nonunions [10]. The shaver and burr were maneuvered very carefully, guided by the tactile sensation of bony tissue and fluoroscopic imaging so as to prevent any kind of damage to the surrounding soft tissues. Reduction of the tuberosity fragment was done with the aid of percutaneously passed pins used as joysticks. Since we were able to achieve good reduction and contact of fragments after freshening the fracture site, we did not feel the need to openly reduce the fracture or perform bone grafting. Percutaneous screws were then passed perpendicular to the fracture line in a divergent configuration. Even though literature suggests incorporation of uninvolved joints to achieve adequate fixation [3,7,8,11], we preserved the subtalar and calcaneocuboid joints because the fracture was already past the osteopenic phase and we believed screw purchase was not a problem. Preservation of uninvolved joints would mean better residual function in an already compromised biomechanical environment. Our minimally invasive approach avoided any wound or skin-related complications. The fracture united uneventfully in five months, and the patient could have a shoeable, painless foot. A comparison of treatment methods for neuroarthropathic calcaneal fractures and their outcomes in some reports is summarized in Table 1.

**Table 1:** Comparison of treatment methods of neuroarthropathic calcaneal fractures and their outcomes in various reports. ORIF: open reduction internal fixation.

Author	No. of cases	Etiology	Management	Outcome
Campbell [3]	1	Diabetic neuroarthropathy	ORIF with subtalar arthrodesis.	Successful arthrodesis achieved but fracture site did not consolidate completely.
Chantelau et al. [4]	12	Diabetic neuroarthropathy	Total contact casting.	Eight cured, three developed osteoarthrosis, one developed a severe deformity requiring custom footwear.
Biehl et al. [6]	Two bilateral calcaneal fractures (four fractures)	Diabetic neuroarthropathy	First: casting in both limbs. Second: ORIF in one limb, casting in other.	Three fractures treated with casting healed uneventfully. One fracture treated with ORIF got infected and was subsequently treated by calcanectomy.
Schon and Marks [8]	Twenty-two hindfoot neuroarthropathic fractures	Multiple	Conservative with casts/braces.	Eleven cured. Eleven required hindfoot fusion due to failure of nonoperative treatment.
Present report	1	Meningomyelocele	Percutaneous screw fixation.	Uneventful union at five months.

Through this case report, we want to highlight our management technique and add to the literature on the management of nonunion of neuropathic calcaneal fracture.

## Conclusions

In conclusion, nonunion of a neuropathic calcaneal fracture is a rare clinical scenario with little guidance available for optimum treatment. Despite the high risk of complications, good functional results and successful limb salvage may be achieved with minimally invasive techniques. Freshening the fracture margins with arthroscopic burr under fluoroscopic guidance followed by percutaneous lag screw fixation seems to be an acceptable treatment option to achieve a well aligned shoeable/braceable foot.

## References

[1] Bruckner FE, Howell A (1972). Neuropathic joints. Semin Arthritis Rheum.

[2] Andersen LB, Dipreta J (2006). Charcot of the calcaneus. Foot Ankle Clin.

[3] Campbell JT (2001). Intra-articular neuropathic fracture of the calcaneal body treated by open reduction and subtalar arthrodesis. Foot Ankle Int.

[4] Chantelau E, Richter A, Ghassem-Zadeh N, Poll LW (2007). "Silent" bone stress injuries in the feet of diabetic patients with polyneuropathy: a report on 12 cases. Arch Orthop Trauma Surg.

[5] Essex‐Lopresti P (1952). The mechanism, reduction technique, and results in fractures of the os calcis. Br J Surg.

[6] Biehl W, Morgan J, Wagner W, Gabriel R (1993). Neuropathic calcaneal tuberosity avulsion fractures. Clin Orthop.

[7] Myerson MS, Edwards WH (1999). Management of neuropathic fractures in the foot and ankle. J Am Acad Orthop Surg.

[8] Schon LC, Marks RM (1995). The management of neuroarthropathic fracture-dislocations in the diabetic patient. Orthop Clin North Am.

[9] Roussignol X (2016). Arthroscopic tibiotalar and subtalar joint arthrodesis. Orthop Traumatol Surg Res.

[10] Schepers T, Patka P (2008). Calcaneal nonunion: three cases and a review of the literature. Arch Orthop Trauma Surg.

[11] Johnson JE (1998). Operative treatment of neuropathic arthropathy of the foot and ankle. J Bone Joint Surg.

